# Potential implications of SARS-CoV-2 epidemic in Africa: where are we going from now?

**DOI:** 10.1186/s12879-020-05147-8

**Published:** 2020-06-15

**Authors:** Carlo Torti, Maria Mazzitelli, Enrico Maria Trecarichi, Owachi Darius

**Affiliations:** 1 Department of Medical and Surgical Sciences, Infectious and Tropical Disease Unit, Magna Graecia University of Catanzaro, 88100 Catanzaro, Italy; 2 Department of Infectious Diseases, Kiruddu National Referral Hospital, Kampala, Uganda

**Keywords:** Coronavirus, COVID-19, SARS-CoV-2, Africa, Low-income countries

## Abstract

The SARS-CoV-2, which emerged from East Asia in December 2019, has rapidly evolved into a global pandemic infecting close to 7 million people. The current uncertainties regarding its impact on Africa calls for critical monitoring of the evolution of the pandemic and correlation of factors that influence the burden of the disease. We herein discuss possible implications of SARS-CoV-2 on the African continent.

## Background

Over the last half-century, more than fifty emerging or re-emerging infectious diseases (EIDs) have occurred worldwide. In Africa alone, almost half of the countries experience an EID epidemic each year. About 75% of these diseases are vector-borne or transmitted via zoonotic agents [1]. International travel is emerging as a crucial facilitator of the rapid spread of these EIDs, as demonstrated by the current coronavirus disease 2019 (COVID-19) pandemic [2–4]. The movement of people between different parts of the globe coupled with the variance in incubation periods for each disease (usually varying between days and weeks) makes it difficult to establish a timely diagnosis of such outbreaks, thus favouring the dissemination of these infections worldwide. This is particularly true for respiratory infections, which are transmitted by micro-droplets or secretions. All these factors contribute to the evolution of pandemics and should be taken into account from a public health perspective.

Towards the end of the year 2019, a new variant of Coronaviruses, the severe acute respiratory syndrome coronavirus 2 (SARS-CoV-2) emerged from East Asia. The SARS-CoV-2 causes respiratory infections of varying disease severity in humans, ranging from asymptomatic to severe cases [5]. The virus is transmitted via respiratory droplets and aerosols from both symptomatic and asymptomatic individuals [6–10]. With international travel facilitating the rapid spread of the virus, many countries including those in Africa adopted syndromic screening at ports of entry as a measure to curb its spread, with evidence showing a delay to the onset of outbreaks by a week [11]. However, syndromic screening has its own shortfalls given its inadequacies at detecting asymptomatic individuals [12].

## SARS-CoV-2 in Africa, a different epidemiologic picture

Following the outbreak of the SARS-CoV-2 infection from East Asia towards the end of the year 2019, the viral respiratory infection has since rapidly evolved into a global health crisis [13]. Within 3 months, the SARS-CoV-2 outbreak had spread to more than 110 countries globally and was officially declared a pandemic by the World Health Organization (WHO) on the 11th of March 2020 [14]. By then, about 120,000 people were infected, with the majority coming from Europe and Asia. As of 8th June 2020 (about 3 months later), the pandemic has infected close to 7 million people from 216 countries, with over 400,000 deaths registered [15].

Yet, as the SARS-CoV-2 pandemic continues to ravage the world with western industrialized countries registering record numbers of deaths in the tens of thousands, the epidemiologic picture in Africa is markedly different. As of June 8th, 2020, only 135,412 cases were confirmed from the African Region, contributing 2% of the total global cases. Mortality attributable to SARS-CoV-2 in Africa stands at less than 1% of the global cases of deaths reported, i.e. 3236 deaths in Africa out of 400,857 global deaths [16].

Scientists remain baffled at the relatively low number of cases in Africa compared to the rest of the world. Could it be that African nations learnt important lessons from previous outbreaks such as Ebola, and thus boosted their surveillance systems to contain the COVID-19 outbreak? When the SARS-CoV-2 virus first arrived in Africa in February 2020, most countries heightened their disease surveillance systems by introducing mandatory screening at ports of entry, setting up isolation and quarantine centres, activating disease surveillance mechanisms from previous Ebola surveillance systems and tracing of contacts of suspects to quickly detect and respond to the outbreak [17]. Other countries went ahead to close airports and impose government lockdown systems as measures to curb the spread of the pandemic. This could partly explain the relatively low number of cases on African soil. The question remains, has Africa done enough in the fight against the SARS-CoV-2?

## Diagnostic challenges in Africa

The global shortage of diagnostic tests for SARS-CoV-2 threatens to undo the progress made by African countries, possibly contributing to an underestimation of the true burden of disease. The WHO currently recommends the use of nucleic acid amplification tests such as the real-time polymerase chain reaction tests (RT-PCR) as the standard for diagnosing SARS-CoV-2 infection [18].

However, PCR tests are often expensive and becoming increasingly inaccessible for most African countries. The level of testing on the continent varies depending on the income status of the countries. South Africa, the second-largest economy in Africa, adopted a mass screening strategy and tests about 16,000 people per day. Other countries adopted a targeted screening strategy with a focus on risk populations that transmit the infection, such as truck drivers in Uganda who contribute > 90% SARS-CoV-2 cases in the country [19]. In the face of scarcity of diagnostic kits, some African nations are shifting their focus to developing cheaper test kits that cost an average of $1 [20].

In an ideal setting, mass screening of the population helps determine the true burden of disease within a country, as demonstrated by South Korea, Germany and South Africa whose mass screening strategies have helped inform their government on strategies to lift lockdowns and implement specific preventive measures [21]. The limited availability of diagnostic tests makes detection of asymptomatic patients near to impossible and deepens the uncertainty of the potential impact of SARS-CoV-2 infection on Africa particularly regarding prevention strategies and effect on economies [7].

## Prevention of SARS-CoV-2 infection in Africa

SARS-CoV-2 is primarily transmitted via respiratory droplets and aerosol particles, spreading from person to person whenever a sick individual coughs, talks or sneezes [22]. Measures such as hand hygiene and social distancing are being advocated as preventive ways to limit the spread of the virus [15, 22]. While these measures are easily applicable to high and middle resource countries, the same cannot be assumed for low resource settings where access to water is still challenging. With a population of 1.3 billion people, it is estimated that 47% of Africans have no access to clean water; a significant bottleneck in preventing the spread of SARS-CoV-2 and other water-borne diseases such as cholera [23]. Emerging evidence demonstrated the isolation of SARS-CoV-2 virus particles in faeces, raising questions over possible faecal-oral route transmission. Although the infectivity of the virus particles isolated in faeces is not yet known, the need to improve sanitation and access to water in Africa has never been greater [24].

Likewise, maintaining a social distance between individuals may not apply in many African countries whose cities are often densely populated with several families living in sharing accommodation and communal settings. Overcrowding and lack of proper spacing have resulted in an increase in the number of people residing in slum settlements. The UN-Habitat estimates that about 200 million people in sub-Saharan Africa live in informal settings, often with poor ventilation facilities [25]. Residents living in such settings are often economically vulnerable, living on a daily wage income to support their dependents [26]. To many of these people, social distancing would be the least of their worries compared to other priorities such as access to food or basic needs. Furthermore, the poor ventilation facilities of these settlements would facilitate the rapid spread of aerosolized infections such as SARS-CoV-2 within the community [9]. These glaring realities threaten to override the preventive measures being advocated for by experts unless population-specific interventions that address the needs of the community are implemented.

The current COVID-19 pandemic is threatening to bring world health systems on the brink of near collapse. There is a worldwide shortage of personal protective equipment for health workers [27] of whom, more than 90,000 have been infected with the SARS-CoV-2 virus globally [27]. In the face of a relentless pandemic, weak health systems and inadequate supplies of personal protective equipment, Africa finds herself in a dire situation. Yet experience from previous infectious disease epidemics should have taught African governments of the need to invest and build their health systems with an emphasis on disease surveillance and careful healthcare planning and financing [28]. Some countries have started manufacturing their own personal protective equipment such as masks and gowns as demonstrated by Kenya [29].

## Vaccines and treatment

To date, no effective vaccine is available to control the SARS-CoV-2 infection despite several ongoing studies. There is currently a drive to produce a SARS-CoV-2 vaccine that could save millions of lives, with experts optimistic of its availability within a year’s time [30]. However, should a vaccine be successfully developed, how rapidly would such an intervention be implemented in Africa?

Majority of the African countries have an extended program for immunization (EPI) through which children below 1 year of age receive vaccinations against common childhood illnesses. The pentavalent vaccine which immunizes children against five common childhood diseases including pneumonia is administered in three doses. Studies, however, show that only half the number of African countries achieved 80% coverage of the third dose of the pentavalent vaccine [31]. This vaccination coverage gap needs to be urgently bridged if indeed a SARS-CoV-2 vaccine is developed in the near future. Furthermore, the current global scramble for personal protective gear highlights the need to ensure equal and fair distribution of life-saving medical resources (such as vaccines) between high and low resource countries [21].

Similar to vaccines, no effective treatment against SARS-CoV-2 infection exists to date. A recent trial showed no benefit in the use of lopinavir/ritonavir, a drug commonly available in the treatment of HIV in Africa [32]. The European AIDS Clinical Society (EACS) and the British HIV Association (BHIVA) stated that there is no evidence to support switching to regimens containing boosted protease inhibitors in order to prevent COVID-19 in people living with HIV [16]. Similarly, there are ongoing studies to determine the efficacy of hydroxychloroquine [17], which previously demonstrated in vitro activity against SARS-CoV-2. However, no study has demonstrated significant mortality benefit nor a consistent clearance of SARS-CoV-2 RNA from respiratory samples with the use of hydroxychloroquine [33–35]. A recent study demonstrated an increased risk of mortality with hydroxychloroquine, attributable to increased risk of cardiac toxicity [36]. Although this paper was withdrawn adding more complexity to the role of hydroxychloroquine, the drug would require consistent cardiac monitoring for toxicity especially when combined with azithromycin. The feasibility of rolling out this treatment in resource-constrained settings where cardiac monitoring may not be readily available makes it an unreliable option. Lastly, a randomised trial demonstrated no significant benefits of hydroxychloroquine used as post-exposure prophylaxis [37], and effects as pre-exposure prophylaxis have only been hypothesised.

Remdesivir (GS-5734), a nucleotide analogue inhibitor of the viral RNA-dependent RNA polymerase of SARS-CoV-2, reduces the time to recovery from a median of 15 days in patients treated with placebo to a median of 11 days in those treated with the drug [38]. Although the study is still ongoing, the authors also found that mortality in the remdesivir group was lower than the placebo arm (7.1% versus 11.9%). Clinical trials are still ongoing to determine the efficacy of remdesivir [38, 39]. However, the intravenous formulation of the drug requiring refrigeration for storage may limit its extensive use in resource-limited settings. It is yet to be determined whether orally available compounds, such as favipiravir (originally used for influenza) or directly acting antivirals (used for hepatitis C infection), could provide any significant effects in patients with COVID-19 [40].

Another promising drug under investigation is tocilizumab, an anti-IL-6 agent used for the treatment of rheumatoid arthritis [41]. COVID-19 is associated with increased production of IL-6 with some reports suggesting some clinical efficacy of tocilizumab when administered intravenously in patients with COVID-19 pneumonia [42–44]. Another report described the efficacy of tocilizumab when administered subcutaneously [45]. This route of administration could be particularly useful in contexts where intravenous administration is not available. However, the availability, cost and monitoring of side effects, as well as the increased risk of superimposed or reactivating infections, may limit the use of tocilizumab and other immune-modulator agents in resource-limited settings.

In contrast to ongoing research in conventional medicine, the role of complementary medicine, such as the use of herbal remedies in treating SARS-CoV-2 infection, has been largely unexplored. In many African settings, herbal and home-made remedies are used by the local population as the first-line treatment for common illnesses such as respiratory infections. It is estimated that about 80% of residents in Africa use herbal remedies for their primary healthcare [46]. Different plant remedies such as leaves, roots or concoctions are used to treat different respiratory ailments such as tuberculosis in Senegal and ear infections in Kenya and Uganda [47–49]. In the absence of effective treatment against SARS-CoV-2 infection, some African countries are exploring the role of herbs in treating the virus such as Madagascar, which recently reported a potential cure of COVID-19 by using a herbal extract derived from an indigenous plant, *Artemisia annua* [50]. However, the lack of scientific evidence to prove the efficacy and safety of the product has raised scepticism among experts. Nevertheless, the unanswered questions regarding the effective treatment of SARS-CoV-2 may provide an opportunity for scientists to conduct scientific studies on herbal remedies in an effort to discover new treatments for this disease.

## Should we expect an exponential increase in SARS-CoV-2 cases and in Africa?

Africa boasts a demographic advantage of having a relatively young population, with a median age of 20 years [51]. With current evidence showing that severe COVID-19 mostly affects people of advanced age associated with noncommunicable diseases [52], the odds of survival appear to favour Africans. Furthermore, the expression pattern of human angiotensin-converting enzyme 2 (ACE2), a receptor for SARS-CoV-2 to gain entry into cells, appears to be low in Africans compared to Asians, further favouring the possibility of positive outcomes [53, 54].

However, Africa has a high burden of infectious diseases and comorbidities such as HIV, tuberculosis and malnutrition, all of which have detrimental effects on the host’s immune system, thus potentially increasing their susceptibility to severe respiratory infections, such as SARS-CoV-2. Although evidence is inconclusive of the potential interactions between SARS-CoV-2 and these comorbidities, it is important to remember that more than 200 million people suffering from malnutrition, 15 million people currently living with HIV and 2.5 million new cases of tuberculosis all reside in Africa. A significant proportion of the African population might be at risk of severe SARS-CoV-2 disease [55–57]. This high burden of infections and comorbidities coupled with weak established health systems sets a platform for the epidemic to spin out of control unless stringent preventive measures are instated. Data on the critical care bed capacity in most African countries is generally sparse [58, 59].

The WHO warns that between 29 and 44 million Africans risk getting infected with SARS-CoV-2 and about 83,000–190,000 people risk losing their lives should the containment measures (such as prompt diagnosis of SARS-Cov-2 infections, contact tracing, isolation, improved personal hygiene and physical distancing) fail [60]. The need for African countries to build and develop their health systems capacity in order to tackle the growing threat of a catastrophic health crisis has never been greater. Previous outbreaks such as Ebola have unravelled the dire need for African governments to invest significantly in disease surveillance, research and strengthening health systems in order to overcome future outbreaks [28]. Last but not least, it is important to consider the One Health approach, which links the health of humans, animals, plants and their shared environments, as an important determinant to control the current SARS-CoV-2 pandemic and its effects. Indeed, the current health emergency highlights the importance of this approach, placing food systems as a key component of One Health actions [61].

## Conclusions

In conclusion, the current uncertainties regarding the impact of SARS-CoV-2 infection in Africa call for critical monitoring of the evolution of the pandemic and factors that influence the burden of disease. Even in the absence of vaccination and effective treatments, Africa can lead the fight against SARS-CoV-2 provided appropriate containment response systems are put in place along with addressing the systematic bottlenecks such as access to water, improvement of food systems, health education, critical care hospital bed capacity and increasing health care financing and investment.

## Data Availability

Not applicable.

## Abbreviations

ACE2: Angiotensin-converting enzyme 2
BHIVA: British HIV association
COVID-19: Coronavirus disease 2019
EACS: European AIDS clinical society
EIDs: Emerging infectious diseases
SARS-CoV-2: Severe acute respiratory syndrome *Coronavirus* 2
WHO: World Health Organization
